# *Gynecologic Oncology Research and Practice*: a new journal to meet the needs of a growing field

**DOI:** 10.1186/2053-6844-1-1

**Published:** 2014-09-30

**Authors:** Robert L Coleman, Thomas J Herzog, Bradley J Monk, Philip Dooner

**Affiliations:** Department of Gynecologic Oncology, University of Texas MD Anderson Cancer Center, Houston, TX USA; Department of Obstetrics & Gynecology, University of Cincinnati Cancer Institute, Cincinnati, OH USA; Creighton University School of Medicine at Dignity Health St Joseph’s Hospital and Medical Center, University of Arizona Cancer Center, Phoenix, AZ USA; BioMed Central, London, UK

## Editorial

With the rapidly expanding field of gynecologic oncology, there is an unmet need to enable the rapid sharing of data. The electronic era now allows speedy review and almost immediate publication of cutting edge scientific and clinical material. Additionally, the opportunity to upload videos, photos and datasets would be a huge advancement for our specialty. All of this in an “open access” format that would be free of charge is sorely needed.

It is with great pleasure that we launch *Gynecologic Oncology Research and Practice* [1], an open access, journal that aims to serve as an international platform for sharing laboratory and clinical findings by offering a high-visibility forum for new insights and discussions among scientists, physicians and oncologists. The journal will publish articles on the pathophysiology, prevention, diagnosis, and treatment of all cancers of the female reproductive system.

Being open access, all articles published in *Gynecologic Oncology Research and Practice* are freely and universally accessible online, so available to readers at no cost with access not limited by their library’s budget. Articles published under this model therefore have the potential to reach a much larger readership than any subscription-based journal [2], with some studies suggesting a correlation between open access, higher downloads and higher citations, leading to a higher Impact Factor [3, 4]. Published articles also comply with the open access policies of major funding bodies, including the Wellcome Trust and NIH [5–8].

The journal is benefited by our high-profile Editorial Board [9] who will aim to deliver a timely, thorough and, above all, fair peer review process. This will not only ensure that authors benefit from a positive publication experience but will guarantee that the journal publishes good quality topical content.

As well as publishing up-to-date research, the journal will also feature topical review articles, interesting cases, novel methodologies, and thought-provoking opinions. Combine this with the option to uploaded unlimited photos, videos and datasets, the ability to post online comments, and the capabilities to immediately share articles via social media, the journal has the potential to become a hub for distributing new knowledge and discussing stimulating topics in the field.

In journal’s inaugural articles, Micael Lopez-Acevedo, Angeles Secord and colleagues explore the activity of dasatinib in combination with gemcitabine and docetaxel in uterine leiomyosarcoma (uLMS) cell lines in order to determine if dasatinib inhibits the SRC pathway [10], a review article by Steven J Gibson and colleagues provides an overview of drug discovery in the ovarian cancer arena [11], and Cassandra D. Foss, *et al*. determine the protein expression profile (PEP) of primary and recurrent ovarian cancer patients in order to predict therapeutic targets for chemotherapy [12].

It is with these thoughts that we welcome new readers and potential collaborators to *Gynecologic Oncology Research and Practice*.
